# Translational and interdisciplinary insights into presbyacusis: A multidimensional disease

**DOI:** 10.1016/j.heares.2020.108109

**Published:** 2021-03-15

**Authors:** Mark A. Eckert, Kelly C. Harris, Hainan Lang, Morag A. Lewis, Richard A. Schmiedt, Bradley A. Schulte, Karen P. Steel, Kenneth I. Vaden, Judy R. Dubno

**Affiliations:** a Medical University of South Carolina, Department of Otolaryngology – Head and Neck Surgery, Charleston, SC 29425, USA; b Medical University of South Carolina, Department of Pathology and Laboratory Medicine, Charleston, SC 29425, USA; c King's College London, Wolfson Centre for Age-Related Diseases, London SE1 1UL, United Kingdom

**Keywords:** Presbyacusis, Age-Related Hearing Loss, Metabolic, Sensory, Neural, Endocochlear Potential, ABR, auditory brainstem response, CF, characteristic frequency, CM, cochlear microphonic, CAP, compound action potential, EP, endocochlear potential, GWAS, genome-wide association studies, HSC, hematopoietic stem cell, IHC, inner hair cells, MBP, myelin basic protein, NF200, neurofilament 200, OHC, outer hair cells, R_HC_, outer hair cells fixed resistance, QDA, quadratic discriminant analysis, Rs, source resistance, SR, spontaneous rate, TEOAE, transient-evoked otoacoustic emissions, ΔR, variable resistance, Vs, voltage source

## Abstract

There are multiple etiologies and phenotypes of age-related hearing loss or presbyacusis. In this review we summarize findings from animal and human studies of presbyacusis, including those that provide the theoretical framework for distinct metabolic, sensory, and neural presbyacusis phenotypes. A key finding in quiet-aged animals is a decline in the endocochlear potential (EP) that results in elevated pure-tone thresholds across frequencies with greater losses at higher frequencies. In contrast, sensory presbyacusis appears to derive, in part, from acute and cumulative effects on hair cells of a lifetime of environmental exposures (e.g., noise), which often result in pronounced high frequency hearing loss. These patterns of hearing loss in animals are recognizable in the human audiogram and can be classified into metabolic and sensory presbyacusis phenotypes, as well as a mixed metabolic+sensory phenotype. However, the audiogram does not fully characterize age-related changes in auditory function. Along with the effects of peripheral auditory system declines on the auditory nerve, primary degeneration in the spiral ganglion also appears to contribute to central auditory system aging. These inner ear alterations often correlate with structural and functional changes throughout the central nervous system and may explain suprathreshold speech communication difficulties in older adults with hearing loss. Throughout this review we highlight potential methods and research directions, with the goal of advancing our understanding, prevention, diagnosis, and treatment of presbyacusis.

## Highlights

• Presbyacusis presents with metabolic, neural, and/or sensory phenotypes.
• Reduced endocochlear potential is likely to be a key contributor to presbyacusis.
• Genetic factors contribute to presbyacusis, including single-gene variants.
• There appear to be downstream effects of presbyacusis on brain function and structure.

## 1 Overview of presbyacusis

### 1.1 The significance and rationale of characterizing human presbyacusis

Hearing loss is among the most common chronic conditions of aging, with a 2-fold higher incidence than cardiovascular disease, a 5-fold higher incidence than diabetes, and a 10-fold higher incidence than cancer (National Academy of Sciences, 2016). The National Institutes of Health reports that approximately 30% of Americans aged 65-74 and 50% of those 75 and older have impaired hearing. When hearing loss at frequencies above 3.0 kHz is included, virtually 100% of the population over the age of 80 has a significant hearing loss. Given that the percentage of people older than 65 will increase by 80% over the next two decades (National Academy of Sciences, 2012), presbyacusis will continue to be a major public health concern.

**Fig. 1:**
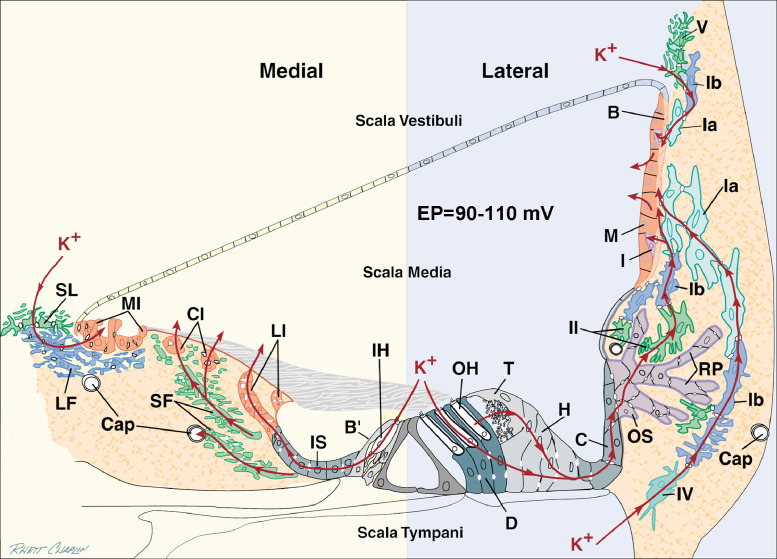
Proposed lateral and medial routes for K^+^ homeostasis. In the lateral route, K^+^ effluxing from outer hair cells (OHC) is resorbed by Deiters (D) and tectal (T) cells and flows via gap junctions through Hensen (H), Claudius (C), and outer sulcus (OS) cells and their root processes (RP) to efflux into stroma maintained at a low K^+^ level by the Na, K-ATPase activity of type II fibrocytes. Other K^+^ ions diffuse into scala tympani and are taken up by type IV fibrocytes. The resorbed K^+^ subsequently diffuses via gap junctions through type I fibrocytes (Ia, Ib) and strial basal (B) and intermediate (I) cells into the intrastrial compartment, which is kept low in K^+^ by the pumping activity of strial marginal (M) cells. K^+^ resorbed by type V fibrocytes from scala vestibuli diffuses downhill through Ib then Ia fibrocytes to the stria. In the medial circuit, ions released from inner hair cells (IHC) diffuse to endolymph through border (B’), inner sulcus (IS) and lateral interdental (LI) cells or pass from IS cells through stellate fibrocytes (SF) to capillaries (Cap) or to central interdental (CI) cells and into the scala media. K^+^ pumped from scala vestibuli into supralimbal (SL) cells flows downgradient to light fibrocytes (LF) and medial interdental (MI) cells for return to scala media. Adapted from Spicer and Schulte, (1998).

Age-related hearing loss in humans is heterogeneous and complex because many factors in addition to aging alone can lead to hearing loss in older persons, such as exposure to noise, ototoxic drugs, or otologic disease (Keithley, 2019; Liberman, 2017; Viana, et al., 2015), as well as genetic contributions. For example, aging and noise trauma that appear to result in outer hair cell (OHC) loss, have additive influences on hearing loss (Wu et al., 2020a, Wu et al., 2020b). In light of these complexities, we and others have studied animals with age-related hearing loss, such as the quiet-aged gerbil, to control the most pertinent variables and allow systematic studies of pathology and physiology at the cellular and molecular levels. Although raised under experimental conditions wherein environmental variables are strictly controlled, these gerbils nevertheless show age-related declines in auditory function. This is consistent with the premise that presbyacusis includes effects attributable specifically to aging and not just the additive effects of noise and other ototoxic factors, and genetic interactions, over a lifetime. A prominent pathologic feature associated with age-related hearing loss in the quiet-aged gerbil is degeneration of the lateral wall of the cochlea (Gratton and Schulte, 1995a; Gratton, et al., 1996; Sakaguchi et al., 1997; Schulte and Schmiedt, 1992; Spicer, et al., 1997; Spicer and Schulte, 2002). The cochlear lateral wall consists of both the stria vascularis and the underlying spiral ligament. Pathological alterations in one or both of these structures can affect potassium (K^+^) recycling and ion homeostasis (Schulte and Steel, 1994; Spicer and Schulte, 1996) and thus the production and maintenance of the endocochlear potential (EP). Potassium recycling is a complex process by which K^+^ effluxed from endolymph through hair cells is actively recycled back to endolymph via a series of intercellular and intracellular pathways (Fig. 1). This process is essential to the maintenance of a robust EP and normal hearing. Comprehensive descriptions of the molecular and physiological basis for generation of the EP and K^+^ recycling are available elsewhere (Hibino and Kurachi, 2006; Schulte, 2008; Wangemann, 2002). In quiet-aged gerbils, pathological changes in the cochlear lateral wall often occur in conjunction with small and scattered losses of OHCs in the most basal and apical regions, with minimal loss in the remainder of the cochlea, and a virtually normal population of inner hair cells (IHCs) (Tarnowski, et al., 1991).

A dramatic feature of age-related hearing loss in animals is variability among individuals. In a study of 36-month-old outbred gerbils with strict control of environmental exposures (Mills, et al., 1990), threshold shifts ranged from 0 dB to > 60 dB. These results and results from different inbred strains of mice (e.g., Zheng, et al., 1999) suggest a strong genetic component in age-related hearing loss, which has been confirmed by relatively high heritability (Demeester, et al., 2010; Gates, et al., 1999; Karlsson, et al., 1997; Viljanen, et al., 2007; Wolber, et al., 2012). This heritability is equivalent to or higher in magnitude than that reported for hypertension (Jensen, et al., 2018) and hyperlipidemia (Williams, et al., 1993). In Section 2, we describe evidence for genetic contributions to presbyacusis and a framework for understanding the genetic underpinnings of presbyacusis.

Understanding genetic determinants and treatments will likely depend on carefully defined presbyacusis phenotypes, as well as comprehensive and specific measures for ascertaining different patterns of cochlear pathology in humans. Section 3 includes findings in laboratory animals documenting atrophic changes in the cochlear lateral wall with age-related degeneration of the strial microvasculature and fibrocytes in the spiral ligament. These and other animal and human temporal bone findings provide the basis for a battery model of metabolic and sensory presbyacusis that is presented in Section 4, including consideration of how noise trauma would influence the cochlear battery and associations with hearing loss. These findings also provide the empirical and conceptual foundations for characterizing presbyacusis phenotypes using human audiograms, as described in Section 5. However, audiometric thresholds are not always predictive of the functional effects of declines in the auditory nerve that could occur independently of age-related metabolic declines. Section 6 addresses these issues, including the consequences of peripheral auditory system degeneration on the auditory nerve and brain. Throughout this review we focus on topics that may advance the field with further study and on methodological approaches to pursue this research.

## 2 Genetics of presbyacusis

### 2.1 Genetic architecture of hearing loss

**Fig. 2:**
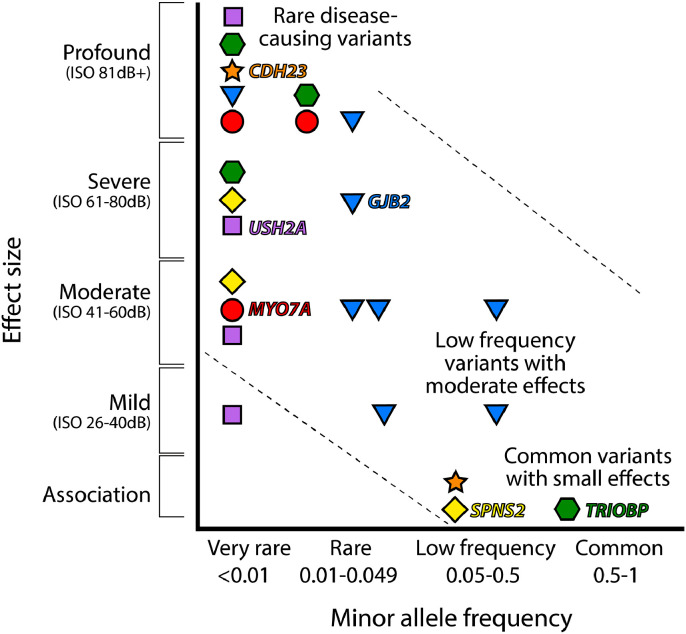
Genetic architecture of disease. This plot of selected genes known to be involved in deafness represents a model where mutations with a large effect size are rare, whereas common diseases are thought to result from the combined effect of many common variants each of which individually has a small effect. Mutations with clinical significance are expected to fall within the band from top left to bottom right. The impact of each variant on auditory thresholds (effect size) is plotted with respect to the frequency of each variant in the population (minor allele frequency). Levels of hearing impairment are clustered into ranges from mild to profound deafness. Variants shown to be associated with hearing level in association studies (Wells, et al., 2019; Ingham, et al., 2019) are plotted at the bottom. Different mutations in the same gene can have different impacts on disease. Rare *MYO7A, TRIOBP, SPNS2, CDH23, USH2A* and *GJB2* mutations can lead to severe or profound deafness (Weil, et al., 1995; Shahin, et al., 2006; Riazuddin, et al., 2006; Ingham, et al., 2019; Bolz, et al., 2001; Kelsell, et al., 1997; Eudy, et al., 1998). Different very rare mutations in *MYO7A* and *USH2A* lead to milder, postlingual progressive hearing loss (Liu, et al., 1997; Molina-Ramirez, et al., 2020), shown by the distribution of *MYO7A* (red circles) and *USH2A* (purple squares) plotted on the left. Relatively common variants in *TRIOBP* (green hexagons), *CDH23* (orange stars) and *SPNS2* (yellow diamonds) have been implicated in hearing loss by association studies (Wells, et al., 2019; Hoffmann, et al., 2016; Ingham, et al., 2019; Molina-Ramírez, et al., 2020), but these genes also are known to underlie profound deafness resulting from very rare mutations. *GJB2* variants (blue triangles) span the spectrum.

Common diseases with adult onset, including presbyacusis, are often considered to be associated with many genetic variants each having a small effect size that add together to lead to the disease, rather than being due to single-gene mutations (Boyle, et al., 2017; Gibson, 2012; Gottesmann and Shields, 1967). However, there are several single-gene mutations that lead to young adult or middle-age onset of progressive hearing loss, mostly dominantly inherited, suggesting that other genetic variants in these genes with milder effects on function could underlie presbyacusis (Kytövuori, et al., 2017; Mencía, et al., 2009; Oonk, et al., 2013). Thirty-nine out of the 44 single genes identified with rare mutations causing dominantly inherited, nonsyndromic deafness lead to progressive hearing loss with a wide range of ages of onset from childhood to 60 years old (references in Van Camp and Smith: www.hereditaryhearingloss.org), supporting a single-gene mechanism for some cases of progressive hearing loss. It is likely that both common variants with small effect size and rare variants with moderate effects are involved in presbyacusis in the population. Furthermore, it is likely that many of the same genes can be involved in both presbyacusis and childhood deafness, making the latter group good candidates for involvement in presbyacusis (Fig. 2). There are several examples of this already known, such as *MYO7A*, which is involved in severe childhood deafness with balance and vision defects (Usher syndrome type 1B) as well as in a milder phenotype - postlingual onset progressive hearing loss (Liu, et al., 1997; Weil, et al., 1995). Recent genome-wide association studies (GWAS) of self-reported hearing loss in the UK Biobank cohort of adults revealed a similar overlap in candidate genes: Wells and colleagues (2019) reported 10 of the 44 associated loci included genes known to be involved in Mendelian childhood deafness, and Kalra and colleagues (2019) found a similar proportion among the 50 significant loci they listed.

Synergistic effects between combinations of genomic variants are likely to contribute to presbyacusis as well as additive effects, and variants can cause enhanced susceptibility to hearing loss triggered by environmental factors. For example, the A1555G variant of the mitochondrial genome makes carriers highly sensitive to the ototoxic effects of aminoglycoside antibiotics (Prezant, et al., 1993), and variants of the *PCDH15* gene have been reported to be associated with increased susceptibility to noise-induced hearing loss (Konings, et al., 2009; Sliwinska-Kowalska and Pawelczyk, 2013; Zhang, et al., 2019).

### 2.2 How can we find genomic variants that predispose to presbyacusis?

The principle method used to identify variants linked to disease in the human population is a GWAS, but in the case of presbyacusis, most studies have failed to identify many, if any, loci of interest. It is important to note that GWAS identify markers linked to a nearby gene that are correlated with a disease rather than a causal mechanism. Thus, GWAS can find common variants with effects on hearing as long as the mutation leading to the variant is ancient, occurring sufficiently long ago that it has had time to become widespread in the population, and can be tagged by close linkage to a suitable marker. However, GWAS cannot detect recent mutations that are not widespread throughout the population [most deleterious rare variants are not widespread (Mathieson and McVean, 2014; Maher, et al., 2012)], and very large sample sizes are usually needed to detect any signal in the noise caused by extensive genetic and environmental heterogeneity.

Unlike GWAS, which relies on markers, exome or genome sequence analysis can find the causative variant(s) for a disease if it lies within the sequenced regions. Exome sequence focusses upon the 2% of the genome that covers the coding regions (exons) so it will often miss regulatory variants that affect mRNA expression levels of genes or the distribution of the resulting proteins in specific cell types, because regulatory regions will very often be in the 98% of the genome outside of the sequence available in exomes. The difficulty with using sequence data is not the detection of variants but assessing the variants for their potential to contribute to presbyacusis. For example, a recent exome sequence analysis of a small set of people with presbyacusis revealed very large numbers of predicted pathogenic variants in genes known to underlie Mendelian deafness (Lewis, et al., 2018); this finding suggests that the pathogenicity predicting algorithms currently used may not be sufficiently stringent. Determination of which variants underlie presbyacusis will require a better control cohort (for example, older adults with normal hearing) and better pathogenicity predictions than we currently have available. Sequence data from family members would be of great help, but is frequently unavailable in the clinical situation where a patient presents with presbyacusis.

As genomic analysis becomes less expensive and cohorts of people with well-characterized auditory phenotypes are assembled, both GWAS and sequence analysis will be useful to build our understanding of the genetic architecture of presbyacusis, providing the information and targets that are needed for development of treatments. We anticipate that these genetic findings will be complemented by human temporal bone studies and validated in mice carrying candidate human variants where the mechanisms and consequences of these genetic variants can be observed.

### 2.3 What has genetics shown us about the pathology of presbyacusis?

The availability and study of large numbers of different hearing-impaired mouse mutants has given us insight into the wide range of pathological processes underlying progressive hearing loss with age (e.g., Ingham, et al., 2019). These correlate broadly with the range of different pathologies reported in temporal bones from people with age-related hearing loss (Schuknecht and Gacek, 1993) and epidemiological observations (Gates and Mills, 2005). Although many hearing-impaired mouse mutants display hair cell defects (corresponding to the sensory phenotype, for example Lewis, et al., 2009; Gibson, et al., 1995), genes contributing to both strial function (metabolic phenotype, for example Ingham, et al., 2016; Ohlemiller, et al., 2009) and innervation (neural phenotype, for example Buniello, et al., 2016) have been identified in this way. All these genes are candidate human deafness genes, and indeed, many have also been linked to human deafness (Mencía, et al., 2009; Weil, et al., 1995; Santos-Cortez, et al., 2016; Buniello, et al., 2016; Kalra, et al., 2019). These mouse mutant and other animal studies have allowed finer definition of the multiple molecular and cellular abnormalities that underlie progressive hearing loss. In the following two sections, we describe histopathologic evidence for presbyacusis from human temporal bone and animal studies, with a focus on age-related anatomical changes that are likely to influence maintenance of the EP (Schmiedt, et al., 2002).

## 3 Non-sensory cells are important for understanding presbyacusis

### 3.1 Fibrocyte loss in presbyacusis

Histopathological analysis of human temporal bones originally classified presbyacusis into four categories, one of which was strial or metabolic (Schuknecht, 1964; 1974). Schuknecht and Gacek (1993) subsequently characterized atrophy of the stria vascularis as the predominant lesion of the aging ear. The term metabolic presbyacusis has gradually replaced strial presbyacusis because animal models have shown that age-related declines in the EP and subsequent hearing loss can result from degenerative changes in the fibrocytes of the spiral ligament, as well as the cells in the stria vascularis (Ding, et al., 2018; Ohlemiller, et al., 2006; Schmiedt, et al., 2002; Schulte and Schmiedt, 1992; Spicer and Schulte, 1998; Wu and Marcus, 2003). Fibrocyte loss appears to be a key feature of metabolic presbyacusis as evidenced by a large reduction in the EP in a mouse model of DFN3 human deafness, which results in the selective degeneration of lateral wall fibrocytes with no pathological changes in the remainder of the cochlea (Minowa, et al., 1999). In addition, mice deficient in the *Otos* gene show elevated thresholds across frequencies and degeneration of lateral wall fibrocytes with no apparent pathology in the stria vascularis or neurosensory epithelium, suggesting a metabolic disorder (Delprat, et al., 2005).

Fibroblast-like cells or fibrocytes, a connective-tissue non-sensory cell type of the inner ear, have been reported to undergo degenerative changes with age in human temporal bones (Kusunoki, et al., 2004). There are at least five types of fibrocytes in the spiral ligament based on their location, morphological features, and histochemical properties (Spicer and Schulte 1991, 1996). Type IV fibrocytes most consistently exhibit age-related pathology. The loss or degeneration of type IV fibrocytes has been reported in gerbils (Spicer and Schulte 2002), CD1 mice (Wu and Marcus 2003), and SAMP8 mice (Menardo, et al., 2012). Type I, II, and/or V fibrocytes have also exhibited age-related pathology in gerbils, CD1 mice, and C57BL/6-Tyrc-2J mice (Spicer and Schulte, 2002; Wu and Marcus, 2003; Menardo, et al., 2012). In addition, spiral ligament fibrocyte degeneration has been observed in C57BL/6 mice before the loss of other cochlear cell types such as hair cells and/or neurons (Hequembourg and Liberman 2001), which suggests that fibrocyte degeneration may be a primary cause of early onset hearing loss in this strain.

Fibrocytes support maintenance of the EP, and specifically inner ear ion and fluid homeostasis, through their role in K^+^ recycling (Fig. 1). The supportive role of fibrocytes in maintenance of the EP was demonstrated in a study where a 7-day exposure to furosemide promoted a significant increase in fibrocyte proliferation, which was associated with a marked recovery of the EP in adult gerbils following the exposure (Lang, et al., 2003). This result is consistent with evidence that fibrocytes in the spiral ligament can repopulate in adult animals and after drug or noise exposure (Lang, et al., 2003; Roberson and Rubel, 1994). Increases in the number of ^3^H-thymidine or BrdU labeled nuclei have been reported in the spiral ligament of gerbils, mice, rats and guinea pigs after acoustic trauma or ototoxin-induced injuries (Roberson and Rubel, 1994; Yamashita, et al., 1999). Unlike sensory hair cells and neurons that have a different developmental origin and are unable to regenerate, fibrocytes continuously turnover in the normal adult cochlea and their increased numbers in response to injury appears to reflect their capacity to proliferate in response to declines in the EP. However, the regenerative ability of fibrocytes appears to decrease with increasing age (Lang, et al., 2003).

Bone marrow is a potential source of stem/progenitor cells responsible for non-sensory cell replacement in the adult cochlear lateral wall (Lang, et al., 2006). Adult stem cells have been identified in a variety of tissues and organs, and they contribute to tissue homeostasis and plasticity by replacement or repair of injured cells continuously throughout life. With increasing age, the self-renewal capacity of stem cells declines, leading to the accumulation of unrepaired, damaged tissues. For example, as hematopoietic stem cells (HSCs) age, there is a marked skewing of HSCs towards the generation of monocytes, red blood cells, or platelets (Liang, et al., 2005). Furthermore, crude bone marrow cells, as well as highly purified HSCs isolated from old mice, do not efficiently generate a specific type of white blood cells (Lang, et al., 2005; Sharp, et al., 1990; Sudo, et al., 2000; Tyan, 1982), and aged human stem cells show a decline in differentiation potential and proliferation rate (Fehrer and Lepperdinger, 2005). These findings suggest the possibility that the age-related decline of fibrocyte turnover in the cochlear lateral wall is due to an age-related decline in the number or function of fibrocyte progenitor cells that derive from bone marrow stem cells.

The links between fibrocyte degeneration and presbyacusis make fibrocytes targets for understanding mechanisms of presbyacusis. However, fibrocyte degeneration may also occur secondarily to pathological changes in the stria vascularis and/or organ of Corti. For example, atypical gap junctions between lateral wall fibrocytes and supporting epithelial cells lining the basilar membrane could disrupt K^+^ recycling (Fig. 1) and then produce fibrocyte and sensory hair cell pathology (Steel, 1999). Additional studies are needed to clearly establish the relationships between fibrocyte degeneration, K^+^ recycling, and age-related hearing loss.

### 3.2 Immunologic (dys)function in presbyacusis

The integrity of the strial microvasculature is needed to maintain the blood-labyrinth barrier and ensure proper function of cells within the stria vascularis, where the EP is generated (Gratton, et al., 1996; Shi, 2016). With aging, the strial microvasculature exhibits pathological changes, such as merged capillaries, thickened basement membrane, and altered transport of charged macromolecules (Gratton, et al., 1997a; Ohlemiller, et al., 2008; Saitoh, et al., 1995, Thomopoulos, et al., 1997). Macrophages, a key element of the innate immune system, regulate vascularization and homeostasis in adult tissues (DeFalco, et al., 2014; Nucera, et al., 2011; Wenes, et al., 2016). Macrophages are present in most regions of the adult cochlea including the organ of Corti (Frye, et al., 2017), the auditory nerve (Kaur, et al., 2015), and the spiral ligament (Hirose, et al., 2005). Macrophages have also been reported to closely interact with the strial microvasculature (Shi, 2016; Zhang, et al., 2013) and are highly sensitive to noise trauma, which can elicit changes in macrophage numbers, morphology, and activation state.

**Fig. 3:**
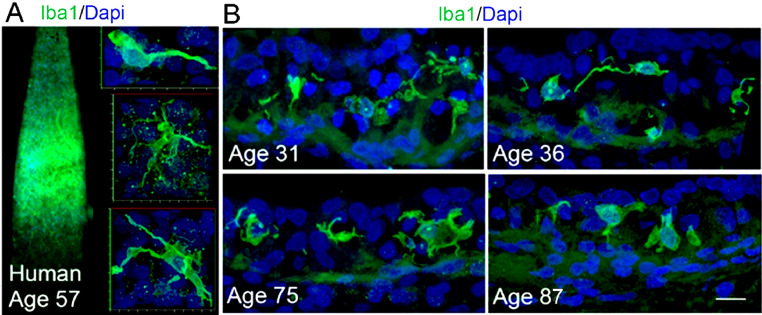
Age-related morphological changes in macrophages in the stria vascularis of human cochleas obtained from temporal bones of human donors. A. Iba1^+^ macrophages have a stellate appearance with thin processes in a whole-mount preparation of the basal cochlear lateral wall from a 57-year-old human donor. B. Strial macrophages from an age-graded series of human temporal bones show age-related alterations. Iba1^+^ macrophages from the two younger donors appear to be in a “surveillance-like” state as characterized by more long processes extending from the central body. In contrast, Iba1^+^ cells from the two older donors often exhibited a rounded shape with increased cytoplasmic volume in the area surrounding the nucleus. Results shown are from Noble, et al., (2019).

Evidence from other organ systems indicates that aging has a profound impact on the function of immune cells, which may result in harmful inflammatory cascades (Oishi and Manabe, 2016; Solana, et al., 2012) and contribute to presbyacusis (Watson, et al., 2017). A report from the Hertfordshire aging study revealed that inflammatory status was associated with elevated audiometric thresholds (Verschuur, et al., 2012). In a recent study (Noble, et al., 2019), temporal bones from younger to middle-aged donors (20-65 years) and older donors (68-89+ years) were processed and examined using the ionized calcium-binding adaptor molecule 1 (Iba1) marker, which is widely used to characterize resident macrophages/microglia (O'Malley, et al., 2016). Morphologic alterations in macrophages in the cochlear lateral wall, along with more activated Iba1^+^ macrophages in this and other cochlear locations, were seen in the inner ears obtained from older donors, as shown in Figure 3 (Noble, et al., 2019). Fibrocytes may also have a role in this pathology as cytokines secreted by macrophages can stimulate fibrocytes to release chemokines that can exaggerate inflammatory responses (Ichimiya, et al., 2000).

Together, age-related alterations in cochlear fibrocytes and macrophages suggest that non-sensory cells in the inner ear play an important role in age-related hearing loss. There is clearly a need to further investigate how inflammation and dysregulation of macrophage activity contribute to strial atrophy in the cochlear lateral wall because the extent of these declines may explain unique patterns of age-related hearing loss. In the following section, we describe how cochlear lateral wall pathology findings have guided the development of a battery model of presbyacusis.

## 4 Basic mechanisms underlying metabolic and sensory presbyacusis

### 4.1 A battery model

The presence of cochlear lateral wall pathology in some older ears has long suggested the existence of a distinct type of presbyacusis that later came to be referred to as metabolic (Schuknecht, 1964, 1974). This became clearer with Sewell, 1984a, Sewell, 1984b, Sewell, 1984c work in acute furosemide-injected cats that demonstrated the strong correlation of the EP in scala media with the characteristics of the cochlear amplifier comprising the OHCs. These characteristics include single auditory-nerve fiber sensitivity at their characteristic frequencies (CFs), overall tuning curve shapes, spontaneous rates (SRs), and rate-level functions. Sewell showed that EP values and the thresholds at CFs of auditory-nerve fibers follow an approximate linear/log curve: a 1 mV drop in EP results in a 1 dB loss of threshold in high-CF fibers. Low-CF fibers are somewhat less sensitive to EP changes (∼2mV/dB). Thresholds of compound action potentials (CAPs) to clicks also show an almost exact 1 mV/dB relation with EP under furosemide application. EP values directly affect the sensitivity of the cochlear amplifier present in the OHC system (Ruggero and Rich, 1991, Schmiedt, et al., 2002, see also Fig. 6A this report).

**Fig. 4:**
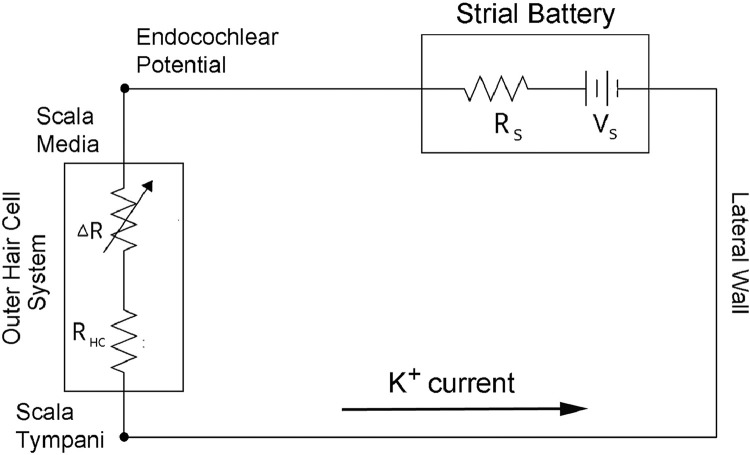
A battery model for understanding interactions between metabolic and sensory presbyacusis. The strial battery is represented by a true voltage source (Vs) and its associated source resistance (Rs). The output of the strial battery is the endocochlear potential (EP) in scala media. The outer hair cell (OHC) system comprises a variable resistance (ΔR) denoting the opening and closing of the OHC cation (K+) channels and a fixed resistance (R_HC_) across the basal membrane of the OHC. Because of the presence of the source resistance, the EP will fluctuate slightly with OHC excitation and inhibition, even in the normal cochlea. This fluctuating voltage riding on the EP is the cochlear microphonic (CM) measured in scala media. Metabolic presbyacusis weakens the strial battery and effectively increases Rs, reducing the EP, yet increasing the CM. In contrast, OHC loss will reduce the K^+^ current from the battery and will effectively increase the EP.

To better understand the physiological basis of metabolic hearing loss and how it may interact with OHC loss, it helps to have a simple electro-anatomic model describing the EP generator and its resulting K^+^ currents (Schmiedt, 1993). The model comprises a battery located across the stria vascularis yielding the EP in scala media (Fig. 4). The battery is coupled to a variable load, the hair cells. Although IHCs certainly draw some current from the strial battery, the OHCs draw about 10 times (20 dB) more, as shown by measurements of the cochlear microphonic (CM) in kanamycin-treated animals (Dallos and Cheatham, 1976). Battery “load” refers to the current obtained from the battery, that is, the greater the load, the greater the current. In reality, it is impossible to have an ideal voltage source, i.e., one where the voltage stays constant no matter what the load resistance. A basic model of a real-world battery is a true voltage source (Vs) with a series resistance internal to the battery, termed the source resistance (Rs). As the battery weakens, the source resistance effectively becomes larger, yielding more voltage drop across it, and less voltage available at the battery output (Ohm's Law):

V = IR

Where V = voltage drop across the resistor in volts, I = current through the resistor in amps, and R = resistance in ohms. In the case of the cochlea, the battery output is the EP.

Given that the scala media is circumscribed by a tight epithelium that isolates it from other cochlear compartments, the circuit describes a battery supplying the EP, which in turn is coupled to a load comprising mainly the OHCs. Obviously, the electro-anatomy of the cochlea is far more complex with both resistive and reactive components (Dallos, 1983, 1984); but a real-world battery feeding the OHC system is a convenient way to visualize some of the consequences of strial pathology and metabolic presbyacusis. It is also clear that a decrease in EP will affect the K^+^ drive through IHCs, though this effect on threshold shift seems far more subtle than that caused by the direct action of the voltage drop on the OHC cochlear amplifier. For example, although neural thresholds can increase by 30 dB or more with chronic EP decrements, rate-level functions of auditory-nerve fibers remain normal in shape at moderate sound levels (Sewell, 1984b; Hellstrom and Schmiedt, 1991). Sewell (1984b) did find that the second, high-level component of the rate-level function was absent with reduced EP. A hypothesis to be explored is that the reduction of the drive current to the IHCs results in the inactivation of the low-SR fibers in the auditory nerve (Liberman, 1978; Schmiedt, et al., 1996).

In this battery model, the battery is connected in series with the OHCs as characterized by a variable resistance (ΔR) representing the cation (K^+^) channels in the stereocilia and a fixed resistance across the basolateral surface of the OHC (R_HC_, Fig. 4). Because the current in the strial battery is predominately K^+^, the return current path to the negative pole of the battery should follow that of the K^+^ recycling pathway (Fig. 1 this report; Schmiedt, 1996; Schulte and Steel, 1994; Schulte, 2008; Spicer and Schulte, 1996; Wangemann, 2002, 2006).

There are several ways the EP can be manipulated in this model. First, the output of the battery can be reduced by increasing the effective source resistance by blocking the active pumps supplying ions to the cochlear lateral wall system. An example is using furosemide to reversibly block the Na, K, Cl cotransporter (Sewell, 1984a). Second, aging can affect battery performance by reducing the density of active pumps in the cell membrane or by reducing the overall pump-containing membrane area in the stria vascularis (Gratton and Schulte, 1995a, Gratton et al., 1995b; 1996; Gratton et al., 1997a, Gratton et al., 1997b; Schulte and Schmiedt, 1992). Third, the model predicts that the EP is modulated by the overall load resistance of the OHC system. If all the OHC cation channels open at once (as in low-frequency biasing) the EP should decline, which it does (Schmiedt, 1982). Conversely, if the channels are all biased closed or if the OHCs have been destroyed by noise exposure, the EP would be expected to be greater than normal, which also is supported (Schmiedt, 1982; Hirose and Liberman, 2003). The CM measured in the scala media can have voltage swings under low-frequency bias conditions greater than 3 mVpp. The loss of OHCs along the cochlear duct will also decrease the load on the strial battery, as discussed below.

### 4.2 The cochlear microphonic as a measurement tool

The rise and fall of the OHC excitation (load) on the EP during sound stimulation effectively results in the CM present within scala media (Dallos, 1973; Schmiedt and Zwislocki, 1977). Thus, the scala media CM can be thought of as a measure of the wellness of the strial battery. That is, a weakened battery with an increased source resistance should result in an *increased* CM. In contrast, if the battery output was a perfect voltage source, there would be no CM riding on the EP in scala media.

**Fig. 5:**
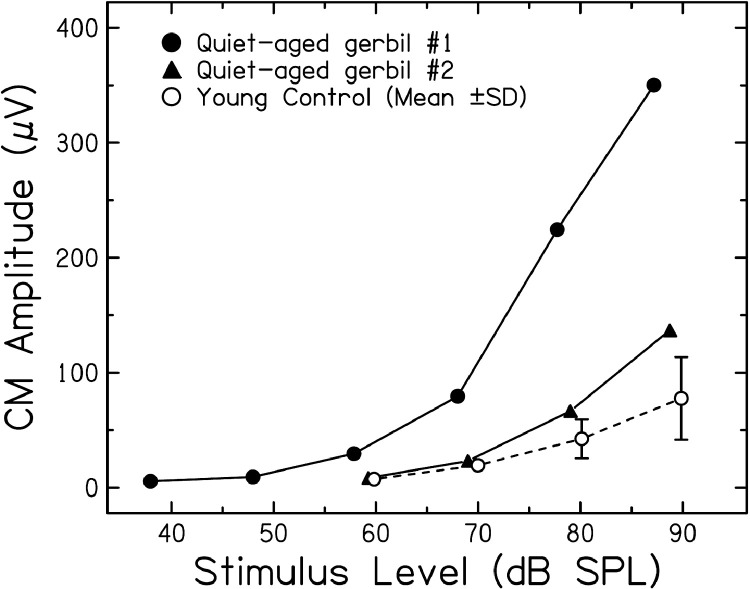
Cochlear microphonic (CM) amplitudes in normal and quiet-aged gerbils as a function of stimulus level. CM was measured near the round window with a compound action potential (CAP) electrode and a voltmeter tuned to the 4 KHz stimulus frequency. The young control values are the mean CM from 9 young gerbils and error bars are one standard deviation. In the two 36 month-old, quiet-aged gerbils shown here, CM was normal at lower levels, and greater than normal at higher levels, despite 30–40 mV decrements in the EP. The larger CM is predicted from the battery model (Fig. 4) with a source resistance (Rs) that is much increased over normal as the result of strial pathologies. Adapted from Schmiedt, (1993).

In metabolic presbyacusis, the strial battery is weakened by fewer active and passive ion channels in the stria vascularis, effectively yielding an increased source resistance and a decreased EP; however, because the voltage fluctuations under a varying load are greater with a higher source resistance, the CM magnitude can *increase* under these conditions. Hellstrom and Schmiedt (1990) and Schmiedt (1993; 2010) show examples of increased CM in some quiet-aged gerbils (Figure 5). When measured with a gross electrode on cochlear bone, these potentials are similar in phase to those measured directly in scala media with micropipettes. Additional data support the hypothesis that strial atrophy coupled with an intact hair cell system can lead to large CM amplitudes (Cheatham, et al., 2011; Kamerer, et al., 2016). Given these results and the predictions of the battery model, measuring the CM in older adults may hold promise as a non-invasive estimate of the health of the cochlear lateral wall. Obviously, these observations are just the beginning of a long process of establishing normative data under varying pathologies before they could be used clinically.

### 4.3 Metabolic presbyacusis and sensory loss: A possible homeostatic interaction

Another example of how the battery model can lend clarity relates to the audiograms of older adults with a history of noise exposure, whose audiometric thresholds at frequencies below ∼2.0 kHz are often near normal in the presence of large losses of basal OHCs (sensory presbyacusis; Dubno, et al., 2013). This is the case even when age would typically suggest some metabolic loss, which would predict more low-frequency hearing loss. Returning to the battery and load model, another way to reduce the load and thereby increase the resting EP, especially with a weakened battery, is by replacing lost OHCs with scar tissue. Under conditions of major basal OHC loss, EP values are predicted to remain near normal with sensory presbyacusis, even with substantial strial pathology.

An important point with regard to this model is that not all regions of the stria are equal in their ability to maintain the cochlear EP. Wu and Hoshino (1999) have shown that minor damage to the stria in the basal turn can substantially change the EP measured more apically in the cochlea, whereas damage to apical stria results in little change of more basally measured EPs. Wu and Hoshino's data suggest that the strial battery is stronger toward the base of the cochlea, and that the location of strial damage should be considered when examining the effects of strial pathology in human temporal bones. Thus, the audiogram of an ear with substantial strial atrophy in the cochlear apex may show a small or no profile of metabolic loss, whereas the audiogram of an ear with moderate amounts of strial pathology in the basal turn may show more evidence of metabolic loss.

**Fig. 6:**
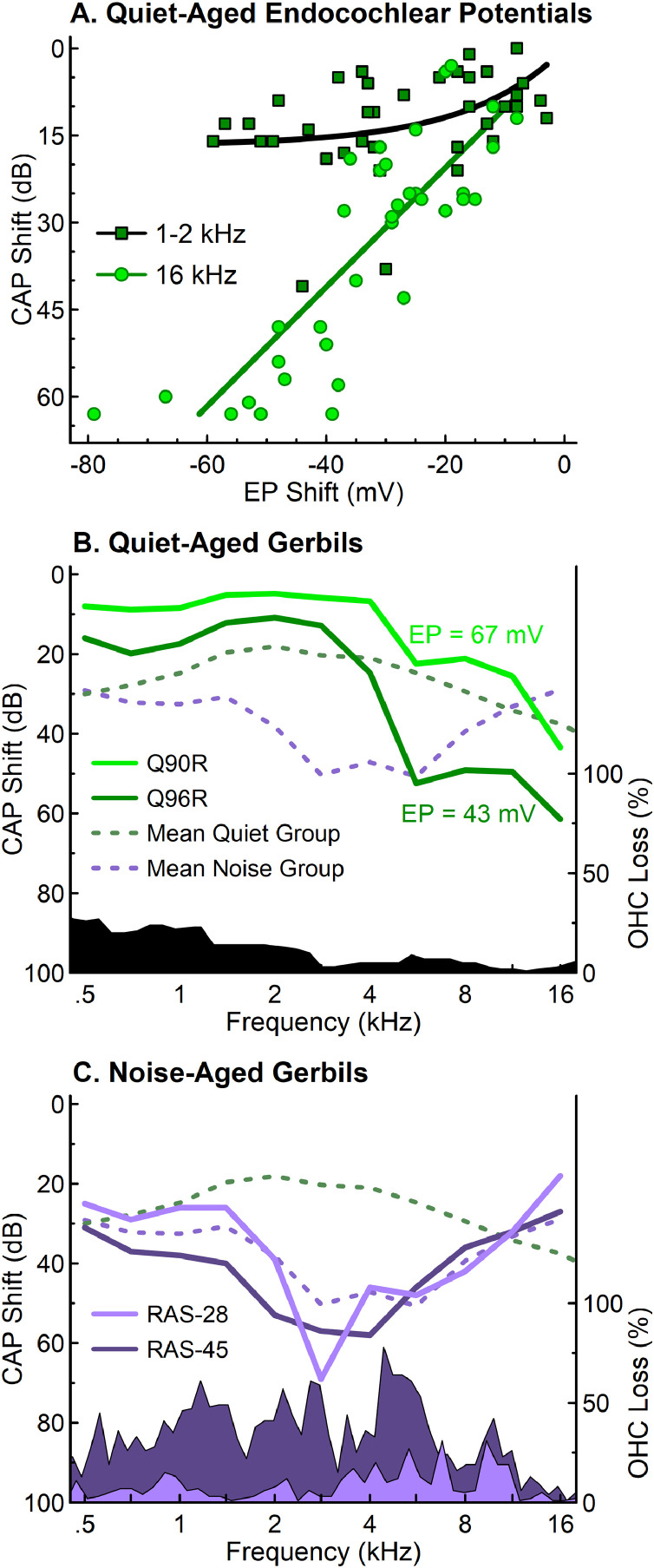
Quiet-aged gerbils exhibited hearing losses that were associated with EP, in contrast with noise-aged gerbils that exhibited significant noise-induced OHC loss. A) EP shifts measured from 35 quiet-aged gerbils were associated with larger CAP threshold shifts at 16 kHz, compared to the 1–2 kHz range where threshold shifts asymptote near 20 dB (Schmiedt et al., 2002). B) Average CAP threshold shifts from 38 to 45 month old quiet-aged gerbils (Schmiedt et al., 2002) illustrated hearing losses that were not well explained by mean OHC losses (black region) in quiet-aged gerbils (Tarnowski et al., 1991). Two representative quiet-aged gerbils (Q90R: 36 months; Q96R: 37 months) showed characteristic minor low frequency hearing loss and sloping threshold shifts above 1.0 kHz (Schmiedt et al., 1990). C) The average CAP threshold shifts for noise-aged gerbils (N = 5; 24–36 months of age) differed from quiet-aged gerbils. Percent OHC losses and CAP threshold shifts for two representative noise-aged gerbils (RAS-28: 24 months; RAS-45: 36 months) reflected the shaped noise field in which each was raised for up to two years (Schmiedt et al., 1990; see text). These results illustrate distinct hearing loss patterns for gerbils due to (1) age-related EP declines, not OHC losses in quiet conditions, and (2) combined age effects and noise-related OHC losses accumulated over a lifetime.

The results of Wu et al. (2020b) detailing hair cell, strial, and neural loss in a cohort of human temporal bones of donors with ages between birth and 104 years demonstrated that audiometric profiles in their cohort of older donors are best correlated with hair cell loss, specifically OHC loss. This result is inconsistent with that found in quiet-aged gerbils, where CAP thresholds had weak to no association with OHC loss (Tarnowski, et al., 1991), but strong associations with EP loss (Schulte and Schmiedt, 1992; see Fig. 6A,B). In addition to the association of audiometric thresholds in older donors with OHC loss, an overarching conclusion of Wu et al. (2020b) is the high prevalence of noise damage in their cohort of human temporal bones, predominately from older male donors, suggesting that noise trauma may be common in the male population in industrialized societies.

How can we reconcile the results from human temporal bone donors with those from quiet-aged gerbils and the battery model of metabolic and sensory presbyacusis? The main difference is that gerbils were raised in quiet with minimal exposure to noise and other ototoxins in order to study pure aging effects, whereas temporal bones from older donors include effects of age and effects of a lifetime of environmental exposures. A better comparison between the animal and human temporal bone data is the experiment of Schmiedt et al. (1990) where 14 eight-month-old gerbils were placed in a reverberant chamber and aged up to 28 months in a wideband noise field (85 dBA, 0.5–4.0 kHz) to examine the effects of a moderate noise exposure over a lifetime. CAP thresholds and OHC counts are shown in Fig. 6C along with averaged threshold data. A similar cohort of quiet-aged control gerbils showed, on average, a small loss of OHCs in the apex and an even smaller loss in the base (Fig. 6B); CAP thresholds showed a metabolic profile as predicted by an age-related reduction in EP and a weak association with OHC loss. In contrast, CAP thresholds of the noise-aged gerbils displayed few signs of metabolic loss, but instead took on the shape of the noise spectrum and the resulting OHC loss (Fig. 6C), similar to the results of Wu et al (2020b) from temporal bones from older human donors.

These results support a homeostatic interaction between metabolic and sensory presbyacusis. When noise exposure ablates or otherwise disrupts OHC function, there will be a higher resistance and less K^+^ current flow across the organ of Corti than in the normal ear with a healthy population of OHCs. Because the stria no longer has to supply as much current as in a healthy cochlea, a normal level of EP may be maintained, despite age-related strial pathologies. Thus, age-related lateral wall pathologies are counterbalanced by OHC loss from accumulated noise exposures. Support for this homeostasis can be found in Hirose and Liberman (2003) where a normal EP was seen in an area of massive strial degeneration with complete OHC loss following acute noise exposure. Another example is seen in Fig. 6C where CAP thresholds at 16.0 kHz for the noise-aged animals are similar to or better than those of the quiet-aged animals. To maintain normal thresholds at high frequencies, a working OHC population *and* a relatively normal EP are necessary. The homeostatic properties of the battery model of presbyacusis are easily tested by measuring EP in quiet-aged and noise-aged animals, using high-pass noise at moderate levels to spare the stria but not basal OHCs. Under these conditions, the battery model predicts higher EPs in noise-aged than quiet-aged animals.

### 4.4 Current injection: A cure for metabolic presbyacusis?

It would seem that if the strial battery is weakened in metabolic presbyacusis and the OHC system remains relatively intact, all that is needed is another voltage source to return the EP to a more normal value and the OHC system to a more normal sensitivity. This is indeed the case as shown by Schmiedt (1993; 2010). Quiet-aged gerbils with reduced EPs ranging from 50–60 mV (compared to the normal value of ∼90 mV) were injected with a K^+^ current via a micropipette into scala media. The current was typically around 10 μA supplied by a constant current generator. CAP thresholds and the maximum amplitudes of the CAP in response to tone pips were measured before, during and after current injection.

As predicted, CAP thresholds became about 10 dB more sensitive across frequencies, and the maximum amplitudes nearly doubled (Schmiedt, 1993; 2010). These results were under conditions of current injections lasting up to an hour. After an hour the EP would precipitously decline and the cochlea became unresponsive. One hypothesis of the origin of the cochlear decline after long-term injection of K^+^ into scala media is that the clearance of the excess K^+^ is limited in these animals. Under normal conditions with a healthy lateral wall, the K^+^ recycling pathway can easily regulate levels of K^+^ in scala media (Fig. 1). If the lateral wall systems are pathologic (as in metabolic presbyacusis), the recycling pathway is almost certainly compromised as well. In short, these results demonstrate the ability to restore hearing thresholds in ears with metabolic presbyacusis by reestablishing the EP, but not necessarily with a chronic injection of a potassium current into the scala media.

Certainly, the future is not one of connecting external batteries to the cochlea. Solutions are likely to be found in the fields of stem cell replacement and genetic therapy, combined with pharmacological treatments to enhance ion pumps and repair the lateral wall systems involved in metabolic presbyacusis. In addition to fibrocytes and intermediate cells in the stria, marginal cells have been shown to be critical in the production of the EP and are affected in older ears. Marginal cell pathology is evident in humans (Schuknecht, 1974) and gerbils (Spicer and Schulte, 2005), as well as in various mouse models, including BALB, CBA/CaJ, *S1PR2*, and Pendrin mutant mice (Royaux, et al., 2003; Ohlemiller, et al., 2006; 2016; Ingham, et al., 2016).

Testing new therapies in humans will depend on careful phenotyping. The following section describes an approach for metabolic and sensory presbyacusis phenotyping in humans using the audiogram that is based, in large part, on the battery model of metabolic and sensory presbyacusis and findings in animals demonstrating unique patterns of histopathology and functional loss in metabolic and sensory loss.

## 5 Human presbyacusis phenotypes

### 5.1 Theoretical framework for unique presbyacusis phenotypes

Based on histopathological evidence from studies of human temporal bones from older adult donors suggesting that metabolic declines were common in these patients (e.g., Bredberg, 1968; Schuknecht and Gacek, 1993; c.f. Wu et al., 2020a, Wu et al., 2020b) and animal studies with distinct patterns of threshold shifts from EP declines or hair cell losses (see Sections 3 and 4; e.g., Mills, et al., 2006; Schmiedt, 2010), a theoretical framework was developed to examine patterns of age-related hearing loss in human audiograms (Dubno, et al., 2013; Schmiedt, 2010). As reflected in the distinct patterns of threshold shifts observed related to EP declines or sensory cell injuries in animal models, discrete phenotypes were predicted for human audiograms along with effects of age, sex, and noise exposure history. Metabolic presbyacusis refers to strial atrophy and EP declines across the cochlear duct that limit hair cell function and were predicted to result in audiograms with mild threshold increases (10–40 dB HL) at lower frequencies, which gradually increase at higher frequencies. Sensory presbyacusis refers to effects of noise or drug exposures, or other injuries (e.g., related to genetic mutations), and were predicted to result in audiograms with a steeply sloping high-frequency hearing loss (≥ 60 dB HL) with normal thresholds at lower frequencies.

Analysis of presbyacusis phenotypes may provide information about how cochlear pathologies affect hearing loss patterns for older adults. To date, the underlying mechanisms of typical metabolic and sensory phenotypes have been difficult to establish based on primate studies or human histopathology (e.g., Engle et al., 2013; Wu et al., 2020b). Wu et al. (2020b) suggested that only OHC loss affects hearing sensitivity. This conclusion was based on results from a clinical sample with mostly occupational noise-exposed older male cases with hearing losses that exceeded standards for otologically normal adults at their ages (International Organization for Standardization; ISO, 2017). This sampling approach differs from community-based studies, which typically include a higher proportion of older female volunteers with fewer reports of excessive noise exposure or noise-related hearing loss compared to males, and include more older adults with normal hearing (Cruickshanks et al., 1998; Dubno et al., 2013; Gates et al., 1990). Audiograms from older adults in large-scale datasets from community-based studies consistently include distinguishable groups with potential metabolic or sensory hearing loss (Allen and Eddins, 2010; Dubno et al., 2013).

### 5.2 Phenotype differences in demographics, pure-tone thresholds, and otoacoustic emissions

Audiometric phenotypes for age-related hearing loss were first established in Dubno, et al. (2013) that evaluated baseline audiograms from 865 adult participants in a longitudinal study (ages 50–89+ years). Each audiogram was classified into one of four phenotype categories based on a Quadratic Discriminant Analysis (QDA) machine learning algorithm: 1) older-normal; 2) metabolic; 3) sensory; or 4) metabolic + sensory. The mixed, metabolic + sensory phenotype included a combination of strial and sensory damage that results in both elevated low-frequency thresholds and steeply sloping high-frequency thresholds (Schmiedt et al., 1990). The results showed that individuals with metabolic and metabolic + sensory phenotypes were significantly older, more frequently female than male, and less likely to report a positive noise history compared to the other phenotypes. The sensory phenotype was significantly younger, more likely to be male, and more likely to report a positive noise history than the other phenotypes. These phenotype differences were consistent with observations in animals, given that metabolic declines occur gradually and may combine with sensory losses (Schmiedt, et al., 1990).

Widespread metabolic declines were also observed by Vaden, et al. (2017), based on audiograms from the same study collected over several years from 343 adults (686 ears), ages 50–89+ years at their first visit. Distinct longitudinal changes were observed for each of the phenotypes (Fig. 6, Vaden, et al. 2017). Pure-tone thresholds increased significantly across frequencies for metabolic and metabolic + sensory phenotypes, whereas high-frequency thresholds significantly increased for the sensory phenotype. Phenotype classifications were consistent for the majority of the ears over an average time period of 5.5 years. Most ears with a changing phenotype transitioned to metabolic or metabolic + sensory phenotypes over an average of 8.2 years. These findings reinforced that metabolic declines are a predominant factor in middle to older adulthood and distinct audiometric profiles are developed over a span of decades (Vaden, et al., 2017).

Because cochlear amplification declines are hypothesized to contribute to metabolic and sensory phenotypes, Vaden, et al. (2018) examined phenotype differences in transient-evoked otoacoustic emissions (TEOAEs) for 656 adults age 50-89+ from the same study. Broad TEOAE declines were observed across frequency bands for metabolic and metabolic + sensory phenotypes, consistent with a lower EP. High-frequency TEOAE declines were observed for the sensory phenotype, consistent with basal OHC damage. The results confirmed that audiometric phenotypes of age-related hearing loss are reflected in the magnitude and configuration of otoacoustic emissions (Vaden, et al., 2018).

### 5.3 Summary of human audiometric phenotypes

Audiometric phenotypes have provided additional support for a theoretical framework with gradual metabolic declines that reduce cochlear amplification and raise pure-tone thresholds, as interpreted through a battery model of metabolic and sensory presbyacusis. Audiograms may contain information about the mixture of metabolic and sensory factors that contribute to an individual's age-related hearing loss. Because future treatment strategies may require diagnostic specificity (Kobrina et al., 2020; Kujawa and Liberman, 2019), it is important to determine how to accurately differentiate sensory and metabolic pathology, including for individuals with more extensive losses in clinical datasets (Parthasarathy et al., 2020) and temporal bone donors (Wu et al., 2020b). The audiogram is not a specific predictor of the functional effects of auditory nerve declines, and in the next section we address evidence from human temporal bone, animal and human electrophysiology/neuroimaging studies that demonstrate the importance of advancing understanding about the consequences of metabolic, sensory, and neural presbyacusis.

## 6 Consequences of peripheral declines and neural presbyacusis

### 6.1 Susceptibility of low-spontaneous rate auditory nerve fibers to inactivity or loss

Auditory nerve fibers in mammals can be classified by their SR (Liberman, 1978; Schmiedt, 1989). High-SR fibers have the lowest CF thresholds, whereas low-SR fibers have higher CF thresholds and are best suited for responding to suprathreshold stimuli. The low-SR fiber population in the gerbil has been shown to be susceptible to EP reduction related to age-related cochlear lateral wall degeneration (Schmiedt, et al., 1996). Functional loss of low-SR fibers is also seen in young gerbils where the EP is chronically lowered for weeks at a time by furosemide (Lang, et al., 2010). These results support the idea that a reduced EP alone can decrease the activity of the low-SR fiber population. The most probable mechanism is that the decreased EP lowers the driving force on K^+^ ions (transduction current), which is needed to depolarize IHCs, to the point where the low-SR fibers are no longer activated. Suryadevara, et al. (2001) have shown in quiet-aged gerbils that the parameter most strongly correlated with spiral ganglion cell size is the EP, in that smaller neurons had lower EPs. Note also that spiral ganglion cell loss in quiet-aged gerbils with metabolic deficits can be substantial; the loss is typically 25% along the entire cochlear duct (Mills, et al., 2006; also see Schulte, et al., 1996) and similar losses are reported in other animal models (Keithley and Feldman, 1979; Keithley, et al., 1989; Keithley, 2019).

It has been shown in some animal species that low-level noise trauma can reduce the numbers of low-SR fibers with minimal threshold shift (Kujawa and Liberman, 2006; 2009; Furman, et al., 2013). Understanding the human condition of “hidden hearing loss” is still evolving (e.g., Kohrman, et al., 2020); however, it is clear from animal studies that EP reduction due to aging or furosemide, primary neural degeneration, and sensory injury alone or in combination can strongly affect the low-SR fiber system.

### 6.2 Age-related myelin pathologies and auditory nerve loss in human temporal bones

**Fig. 7:**
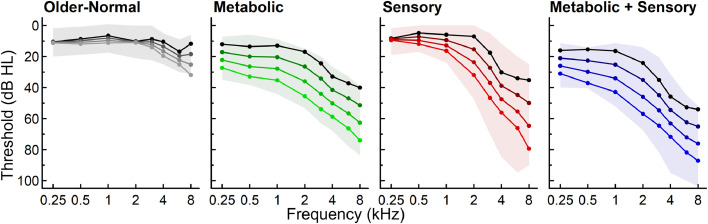
Distinct patterns of age-related hearing loss for each of four audiometric phenotypes. The shaded regions show pure-tone threshold ranges for expert-rated audiograms used to train the Quadratic Discriminant Analysis (QDA) classifier. A regression model was used to predict longitudinal pure-tone thresholds for each phenotype at ages 55, 65, 75, and 85 years old (lighter shades indicate older ages). Adapted from Figs. 1 and 3 in Vaden, et al., (2017).

**Fig. 8:**
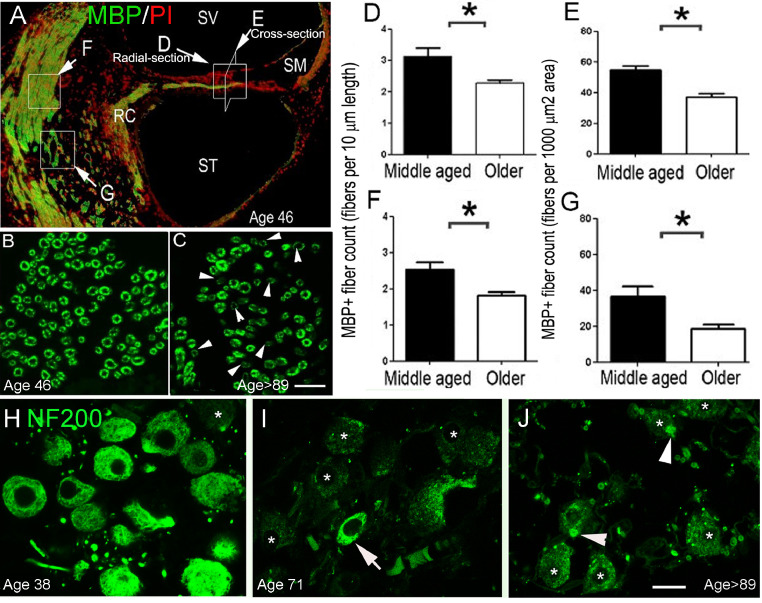
Age-related auditory nerve loss and demyelination. Myelin basic protein (MBP+) nerve fibers (green) are shown in central portions of auditory nerves from a 46-year-old (A, B) and 89+-year-old (C) donor. Losses of MBP^+^ fibers and decreased MBP immunoreactivity in remaining myelinated fibers (white arrowheads) were seen in older donors. White boxes with associated letters (white arrows) in A indicate regions of auditory nerve that were counted in D–G. Counts of MBP+ fibers in peripheral (D, E) and central (F, G) processes of the auditory nerve from the middle turns revealed statistically significant reductions in fiber density in older (n = 5) compared to middle-aged (n = 4) human cochleas (*p < 0.05). (H–J) Images of spiral ganglion neurons in the middle cochlear turn stained for a neuronal structural protein, neurofilament 200 (NF200). The specimens were obtained from middle-aged (H) and older (I, J) donors. There was marked reduction in the intensity of immunostaining for NF200 in most spiral ganglion neurons in the older donors compared to the middle-aged donor. Note the punctuate inclusions (white arrowheads) in the cytoplasm of some NF200+ neurons. Scale bar, 12 µm in C (applies to B, C); 7 µm in J (applies to H–J). RC, Rosenthal's canal; SM, scala media; SV, scala vestibuli. Adapted from Xing, et al., (2012).

About 95% of auditory nerve fibers in a variety of mammals, including human, are myelinated by Schwann cells (Thomsen, 1967; Kiang et al., 1982, 1984; Berglund and Ryugo, 1986; Romand and Romand, 1987), although some somas of human spiral ganglion neurons are unmyelinated (Tylstedt, et al., 1997; Glueckert, et al., 2005). Myelination of the auditory nerve and proper function of myelinating glial cells are critical for normal auditory nerve function, as well as injury repair (Alizadeh, et al., 2015; Wan and Corfas, 2017). Disrupted glial function and myelin deficiency significantly degrade auditory nerve function (Jyothi, et al., 2010; Kurioka, et al., 2016; Panganiban, et al., 2018; Rattay, et al., 2013; Wan and Corfas; 2017; Zhou, et al., 1995). Myelin degeneration is also a key feature of neural pathology in aged animals (Cohen, et al., 1990; Hoeffding, et al., 1988; Xing, et al., 2012). In a study of 20 temporal bones from human donors between 38 and 89+ years of age (Fig. 7), immunostaining of myelin basic protein (MBP) was present throughout the auditory nerve (Xing, et al., 2012). Significant reductions in auditory nerve MBP immunoreactivity (Fig. 8A–C) and MBP+ fiber number were observed in the cochlea of older donors (Fig. 8D-G), as compared to middle-aged donors (Xing, et al., 2012). A reduction of neurofilament 200 (NF200) immunoreactivity, a neurofilament marker used for qualitative and quantitative analysis of spiral ganglion neurons (Roman, et al., 1990; Jyothi, et al., 2010; Kaur, et al., 2018), also revealed structural defects in surviving neurons in the tissue from older donors, as shown in Fig. 8H–J (Xing, et al., 2012). These results are consistent with previous human temporal bone studies showing age-related loss of spiral ganglion cells (Kaur, et al., 2020; Makary, et al., 2011). In addition, myelin degeneration and myelinated fiber loss identified in the cochleas of the older donors suggests that dysfunction of myelinating Schwann cells may contribute to auditory nerve pathology and the subsequent decline of auditory function in neural presbyacusis.

### 6.3 Age-related changes in human auditory nerve function

**Fig. 9:**
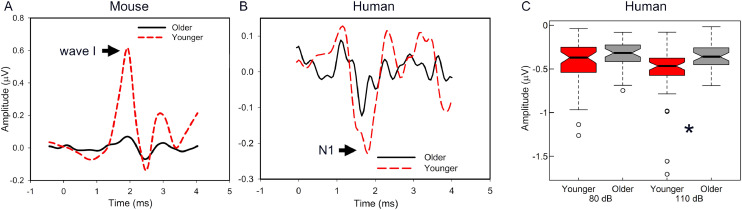
Age-related differences in auditory nerve function across species. A. ABR Wave I was smaller in older compared to younger CBA/CaJ mice (11.0 kHz tone-burst, 85 dB SPL). B. CAP N1 was smaller in older compared to younger adult human subjects (100 µS click, 110 dB pSPL). C. Amplitude differences in N1 in B were also observed in another group of younger and older adults where age-related differences were observed at signal levels of 110 dB pSPL (* *p* < 0.001) but not at 80 dB pSPL (McClaskey, et al., 2018), which further suggests that age-related declines in the amplitude of the N1 are level-dependent.

Auditory nerve function can be characterized in humans using the N1 of the CAP or the equivalent Wave I of the auditory brainstem response (ABR; Burkard and Sims, 2001). These extracellular potentials provide a relatively gross representation of the summed response from a population of auditory nerve fibers. The levels of stimuli needed to evoke these summed responses (CAP and ABR thresholds) are often elevated in the presence of pronounced cochlear damage but may be less sensitive to partial neural degeneration that is likely to occur in older adults (Liberman and Dodds, 1984; Schuknecht, 1953). However, CAP/ABR Wave I responses to stimuli at suprathreshold levels may provide novel information pertaining to auditory nerve function and integrity, including neural degeneration. In animals, where neural loss can be quantified, a partial loss or dysfunction of IHC synapses, spiral ganglion, or auditory nerve fibers results in reduced maximum amplitudes and shallower slopes of amplitude input-output functions (Hellstrom and Schmiedt, 1990; Nadol, 1997, Mills and Schmiedt, 2004, Kujawa and Liberman, 2015). Cross-sectional data from humans suggest a similar phenomenon, with older adults showing shallower slopes of Wave I amplitude input-output functions and reduced maximum amplitudes compared to younger adults (Konrad-Martin, et al., 2012; McClaskey, et al., 2018). Similar age-related differences in auditory nerve function are observed in rodents, as shown in Figure 9. Longitudinal studies of the CAP/ABR, perhaps including single trial and/or multi-metric approaches (Harris et al., 2018, Huet et al., 2019), are needed to characterize the rate of age-related change in these measures.

Studying neural presbyacusis in humans can be difficult because peripheral auditory system declines may affect auditory nerve function. These effects can be addressed by comparing response amplitudes from younger and older adults with relatively normal pure-tone thresholds. Burkard and Sims (2001) and McClaskey, et al. (2018) used this sampling approach and found significantly smaller CAP amplitudes in older compared to younger adults, consistent with a reduction in the maximum amplitude of the CAP Wave I with advancing age that is independent of threshold elevation (Fig. 9). Similar reductions in ABR Wave I amplitudes have been reported in a large cohort of predominantly male military veterans who were 26 to 71 years of age with a wide range of hearing thresholds, where increased age was related to substantially lower amplitudes of ABR Wave I, and these amplitude differences were largely independent of hearing thresholds (Konrad-Martin, et al., 2012). Although our previous assessment of longitudinal changes in pure-tone thresholds and noise history in humans did not show a significant interaction between age and noise history effects (Lee, et al., 2005), animal studies suggest a synergistic effect of age and prior noise exposure, with prior exposure leading to greater age-related auditory nerve loss than in quiet-aged animals (Fernandez, et al., 2015, Kujawa and Liberman, 2015). Longitudinal studies are needed to characterize interactions between age and noise exposure on auditory nerve function in humans.

Acoustic information is encoded by differences in the spike timing and rates across auditory nerve fibers. The redundancy of afferent information coded in auditory nerve fibers means that a loss of as much as 80% of auditory nerve synapses can occur without affecting detection of pure-tone signals (Lobarinas, et al., 2013; Schuknecht and Woellner, 1953). However, loss of these fibers is thought to contribute to an under-sampling of the auditory signal (Lopez-Poveda and Barrios, 2013), among other effects. Although this under-sampling would not affect detection thresholds, it may impact neural encoding of higher level or rapid onset signals, particularly in complex listening environments when decoding is dependent on the resolution of the auditory system. Deficits in auditory nerve function have been associated with deficits in auditory processing and poorer speech recognition in challenging listening conditions (Bramhall, et al., 2018; Harris, et al., 2018; Marmel, et al., 2015). For example, Bramhall, et al. (2018) in a cross-sectional study reported in a sample of 57 subjects that ABR Wave I amplitudes decreased with increasing age, and that decreased Wave I amplitudes and hearing loss were associated with poorer speech recognition in noise. In contrast, studies limited to younger and middle-aged adults have largely failed to find an association between ABR Wave I amplitudes and speech-in-noise recognition (Guest, et al., 2018). Although additional work is necessary to understand the specific effects of auditory nerve loss on speech recognition, age-related deficits in auditory nerve function likely contribute to additive declines in function and structure throughout the auditory system.

### 6.4 Age-related hearing loss associations with brain structure

Age-related inner ear declines are thought to initiate changes throughout the central auditory system and perhaps across other neural systems. Evidence for these widespread effects in humans includes associations between hearing thresholds and brain morphology. A close examination of these findings and their putative mechanisms is important because some suggest that these findings can explain age-related changes in speech recognition, as well as associations between elevated pure-tone thresholds and lower general cognitive function (Slade, et al., 2020 for review). Here we explain why caution is warranted when considering brain structure correlations as evidence for a hypothesized relationship between hearing loss and general cognitive function.

Associations between pure-tone thresholds and auditory cortex gray matter volume have been reported in multiple cross-sectional studies [(Eckert, et al., 2012; Husain, et al., 2011; Peelle, et al., 2011; Ren, et al., 2018; Rigters, et al., 2017; Schneider, et al., 2009); but not in others: (Alfandari, et al., 2018; Boyen, et al., 2013)]. Findings of reduced gray matter volume and cortical thickness appear to be more pronounced for high-frequency compared to low-frequency pure-tone thresholds (Eckert, et al., 2012; Eckert, et al., 2019; Schneider, et al., 2009). These high-frequency threshold observations are consistent with evidence that noise-induced hearing loss had an effect on the morphology of neurons and supporting tissue in rodent auditory cortex (Saljo, et al., 2002; Su, et al., 2017), as well as evidence that sudden and/or unilateral hearing loss was correlated with lower gray matter volume in human auditory cortex (Wang, et al., 2016; Yang, et al., 2014).

It is tempting to interpret the hearing loss and brain morphology findings as reflecting the consequences of sensory presbyacusis. However, it is possible that inner ear and auditory cortex morphology are independently affected by age-correlated factors. This perspective is supported by evidence that the strength of associations between hearing thresholds and primary auditory cortical thickness was similar to the strength of associations between hearing thresholds and primary motor and somatosensory cortical thickness (Eckert, et al., 2019). Moreover, statistically significant increases in low- and high-frequency thresholds did not appreciably correlate with statistically significant changes in regional brain volume over 2.62 years across middle-aged to older adults (mean age at baseline = 64.12 ± 10.32 years), even though cross-sectional significant associations between auditory cortex gray matter volume and high-frequency hearing loss were observed at each time point (Eckert, et al., 2019).

**Fig. 10:**
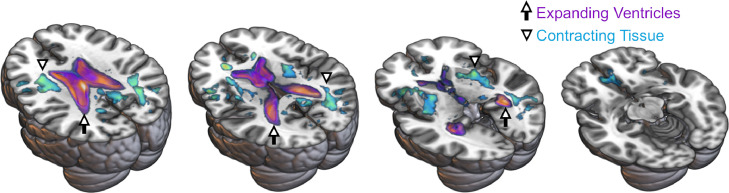
Baseline high-frequency hearing loss was associated with greater longitudinal increases in lateral ventricle volumes measured in 30 middle-aged and older adults who were studied over a span of 2.62 years. Arrows highlight the purple-orange clusters where these effects occurred. Longitudinal contraction of white matter, perhaps due in part to axonal and/or myelin loss, was not significantly related to high-frequency thresholds at baseline after multiple comparison correction. However, location of these uncorrected and medium effect size white matter results shown in the teal clusters suggests that diffuse, and perhaps spatially non-overlapping, white matter declines contributed to the ventricle results. Arrowheads highlight the teal clusters where these volumetric effects occurred. Results from Eckert, et al., (2019) are shown as Cohen's *d* effect sizes (medium *d* = 0.5 to large *d* = 2.0) on the Montreal Neurologic Institute 152 template brain.

An additional interesting finding from the Eckert, et al., (2019) longitudinal study, as shown in Fig. 10, was that elevated high-frequency thresholds at baseline were associated with greater longitudinal expansion of the lateral ventricles. This result is broadly consistent with evidence that middle-aged to older adults with hearing loss at the beginning of a longitudinal study exhibited greater declines in total brain volume in comparison to those without hearing loss (Lin, et al., 2014). Together, these findings suggest that inner ear declines precede subsequent declines in brain structure. Longitudinal studies with multiple measures over many more years may be necessary to observe gradual, age-related hearing losses and subsequent changes in relatively gross brain structure measures like gray matter volume. The challenges of running a long-term longitudinal study are compounded by the considerable functional plasticity demonstrated by the central auditory pathway (Caspary, et al, 2008; Shang, et al., 2018) and perhaps common underlying causes of cortical and inner ear declines (e.g., TRIOBP genetic variant associations with inner ear function and structure: Hoffmann, et al., 2016; Kitajiri, et al., 2010; Yan, et al., 2016; and brain: Vojinovic, et al., 2018). These additive factors likely contribute to the complexity of understanding presbyacusis-mediated declines in brain structure.

The significance of the gross brain structure and hearing associations to age-related differences in speech communication and cognition also remains unclear. Expanded lateral ventricles have been related to lower general cognitive function in people whose post-mortem brains exhibited lacunar infarcts, neuritic plaques, and/or cortical alpha-synuclein pathology (Erten-Lyons, et al., 2013). Relatively normal general cognitive function has been an inclusion criterion for many of the studies in which hearing loss and brain structure associations have been observed. However, it is possible that older adults with the most pronounced hearing loss and evidence of relatively more advanced brain aging are at greatest risk for subsequent impaired speech communication and impaired general cognitive function (e.g., structural predictors of future cognitive declines in Alzheimer's disease: Burggren and Brown, 2014). This could be due to direct effects of presbyacusis, as well as possible common causes that produce declines in both the inner ear and the brain.

## 7 Overall summary

The importance of identifying the different mechanisms and consequences of presbyacusis is underscored by diminished quality of life for older adults with hearing loss and their family members. Here, we have described different presbyacusis phenotypes that have distinct patterns of audiometric expression with specific pathophysiology identified in animals. Differentiating these subtypes of age-related hearing loss may lead to development of tailored forms of intervention to optimize healthy aging, perhaps through targeting genetic mechanisms contributing to declines and protection from declines.

There are multiple mechanisms for hearing loss due to genetic mutations that are expressed with aging and environmental exposures, including noise trauma, that can affect different functions of the inner ear. In humans, these factors are likely additive and perhaps interacting to create a complex audiologic phenotype. This premise is supported by parallel findings in animals and human data demonstrating age-related hearing loss that occurs with a modestly sloping audiogram with loss across frequencies can be explained by age-related pathology of non-sensory cells in the cochlear lateral wall with attendant changes in K+ recycling mechanisms and reductions in the EP. These changes deprive the cochlear amplifier of its essential power supply and promote a loss or inactivity of low-SR auditory nerve fibers. In addition, hearing loss is often related to noise exposure and OHC loss, particularly in males with elevated high frequency thresholds. A similar audiometric pattern can be seen in animals with hair cell loss after exposure to noise or ototoxic drugs. Variation in the sensory phenotype may be dependent on genetic interactions that either increase susceptibility or provide protection to/from noise and may be associated with effects throughout the auditory system. It is also possible that genetic mutations contribute to the loss of hair cells and produce the sensory presbyacusis phenotype in humans.

Audiometric measures of pure-tone thresholds do not fully reflect the functional consequences of age-related pathology of the inner ear. Primary neural degeneration, as well as secondary neural declines due to EP loss and/or synaptopathy and hair cell loss, contributes to declines in auditory nerve function. These declines, which appear to include glial cell pathology and demyelination, likely contribute to the communication difficulties that older adults experience. New approaches for measuring and analyzing the CAP show promise in characterizing the function of the auditory nerve, including perhaps the ability to differentiate the health of low-SR fibers that may be particularly vulnerable to effects of aging.

The primary remedial options currently available for presbyacusis are devices such as hearing aids and cochlear implants, which may provide some benefits but do not restore normal function. There is a large unmet need for biologic approaches to slow or reverse progressive hearing loss, or delay its onset. Developing effective treatments will require knowledge of the molecular and cellular basis of presbyacusis, as well as functional metrics that are sensitive to presbyacusis phenotypes to diagnose and track treatment outcomes.

## Author contributions

Mark A. Eckert: Conceptualization; Judy R. Dubno, Mark A. Eckert, Kelly C. Harris, Hainan Lang, Morag A. Lewis, Richard A. Schmiedt, Bradley A. Schulte, Karen P. Steel, Kenneth I. Vaden, Jr.: Writing - Original Draft; Judy R. Dubno, Mark A. Eckert, Kelly C. Harris, Hainan Lang, Morag A. Lewis, Richard A. Schmiedt, Bradley A. Schulte, Karen P. Steel, Kenneth I. Vaden, Jr.: Writing - Review & Editing

## Declaration of Competing Interest

None.
